# GM-CSF: Orchestrating the Pulmonary Response to Infection

**DOI:** 10.3389/fphar.2021.735443

**Published:** 2022-01-17

**Authors:** Thomas S. McCormick, Rana B. Hejal, Luis O. Leal, Mahmoud A. Ghannoum

**Affiliations:** ^1^ Center for Medical Mycology, Department of Dermatology, Case Western Reserve University, Cleveland, OH, United States; ^2^ Medical Intensive Care Unit, University Hospitals Cleveland Medical Center, Cleveland, OH, United States; ^3^ Pulmonary and Critical Care Medicine, Case Western Reserve University, Cleveland, OH, United States; ^4^ Partner Therapeutics, Lexington, MA, United States; ^5^ University Hospitals Cleveland Medical Center, Cleveland, OH, United States

**Keywords:** GM-CSF, alveolar macrophage, granulocyte-macrophage colony-stimulating factor, sargramostim, COVID-19, acute respiratory distress syndrome, immune response, respiratory infections

## Abstract

This review summarizes the structure and function of the alveolar unit, comprised of alveolar macrophage and epithelial cell types that work in tandem to respond to infection. Granulocyte-macrophage colony-stimulating factor (GM-CSF) helps to maintain the alveolar epithelium and pulmonary immune system under physiological conditions and plays a critical role in restoring homeostasis under pathologic conditions, including infection. Given the emergence of novel severe acute respiratory syndrome coronavirus 2 (SARS-CoV-2) and global spread of coronavirus disease 2019 (COVID-19), with subsequent acute respiratory distress syndrome, understanding basic lung physiology in infectious diseases is especially warranted. This review summarizes clinical and preclinical data for GM-CSF in respiratory infections, and the rationale for sargramostim (yeast-derived recombinant human [rhu] GM-CSF) as adjunctive treatment for COVID-19 and other pulmonary infectious diseases.

## 1 Introduction

The emergence of severe acute respiratory syndrome coronavirus 2 (SARS-CoV-2) as a global health crisis emphasizes the importance of understanding the coordination and crosstalk between alveolar epithelial cells (AECs) and alveolar macrophages (AMs) in maintaining lung physiology during viral illness (Lang et al., 2020). Granulocyte-macrophage colony-stimulating factor (GM-CSF) is an immune-modulating cytokine that plays a critical role in maintaining the alveolar epithelium and pulmonary immune system under homeostatic and pathologic conditions, including infection (Rosler and Herold, 2016; Lang et al., 2020). GM-CSF drives AM differentiation and homeostasis and maintains the integrity of the lung epithelium under near-constant exposure to inhaled pathogens (Trapnell and Whitsett, 2002; Rosler and Herold, 2016). In response to external insult, GM-CSF facilitates and accelerates the epithelial wound-healing process, driving the immune functions of the AMs and the repair processes initiated in the alveolar epithelium, including restoration of barrier function, and facilitating a return to homeostasis (Rosler and Herold, 2016).

## 2 Functions of the Alveolus

### 2.1 Maintaining Alveolar Homeostasis

**FIGURE 1:**
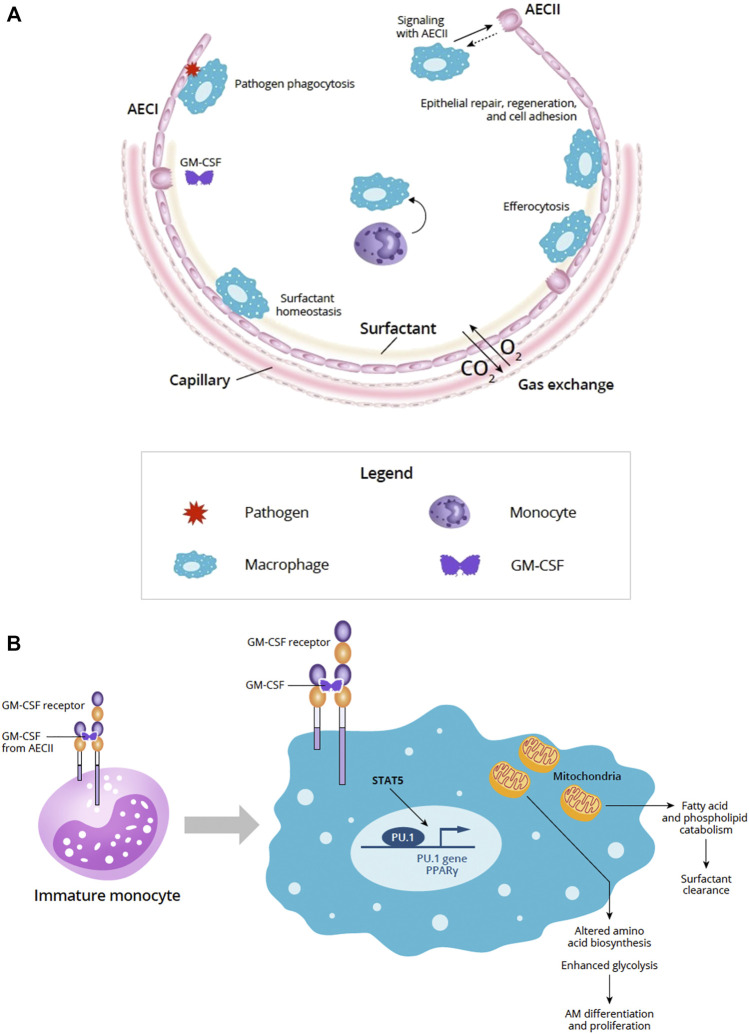
**(A)** Overview of the alveolus under homeostatic conditions (Adapted from Guillot et al., 2013). The alveolar epithelium is important for gas exchange and protection from pathogens *via* the physical epithelial barrier and phagocytosis. Alveolar homeostasis involves the orchestration of multiple cell types including AECI and AECII and AM. Signaling between AECs and AMs is important for epithelial repair, regeneration, and cell adhesion. AECII produce GM-CSF and other cytokines that participate in key homeostatic processes, such as surfactant homeostasis by AM. AM-driven efferocytosis of epithelial or immune cells undergoing apoptosis also helps to maintain the alveolar environment. **(B)** Role of GM-CSF in AM maturation and GM-CSF signaling in AM and mitochondrial mechanisms. In immature monocytes, upon binding to its receptor, GM-CSF signaling through JAK2 and STAT5 activates PU.1 transcription factor initiating differentiation into mature AM. In mature macrophages, GM-CSF signaling through JAK2 and STAT5 activates PU.1 and PPARγ transcription factors and affects the AM transcriptome and metabolism. Effects of these changes in AM mitochondria can result in altered amino acid biosynthesis and enhanced glycolysis needed for AM differentiation and proliferation. AM mitochondrial changes also support fatty acid and phospholipid catabolism required to support surfactant clearance. Abbreviations: AECI, alveolar epithelial cell type I; AECII, alveolar epithelial cell type II; AM, alveolar macrophage; CO_2_, carbon dioxide; DC, dendritic cell; GM-CSF, granulocyte-macrophage colony-stimulating factor; O_2,_ oxygen; STAT5, signal transducer and activator of transcription 5.

The alveolar epithelium is essential for gas exchange in the lung and serves as a physical barrier between the lumen and the underlying submucosa (Guillot et al., 2013). The alveolar epithelium accounts for 99% of the surface area of the lung and is continually bombarded by external insults, such as inhaled particulates and pathogens (Guillot et al., 2013). Maintaining alveolar homeostasis and protecting the integrity and function of the epithelial layer requires the dynamic orchestration of multiple cell types, including type I and type II AEC (AECI, AECII, respectively) and alveolar macrophages, as well as processes such as repair and regeneration, surfactant homeostasis, and pathogen clearance and defense (Figure 1A) (Fehrenbach, 2001; Mason, 2006; Guillot et al., 2013).

Maintaining the integrity of the alveolar epithelium is a key function of alveolar tight junctions that seal the lateral space between adjacent AECs and control the passage of molecules between the alveolar lumen and interstitial compartment to help defend against infection (Guillot et al., 2013; Short et al., 2016; Wittekindt, 2017). Pathogens that target the alveolar epithelium can interfere with tight junctions; for example, respiratory viruses such as influenza have evolved mechanisms to disrupt or alter tight junctions (Short et al., 2016). *Pseudomonas aeruginosa* has also been shown to cause disruption of tight junctions in patients with cystic fibrosis (Higgins et al., 2016), and *Aspergillus fumigatus* proteases damage the respiratory epithelial barrier, leading to the loss of tight junctions (Knutsen et al., 2002).

### 2.2 Alveolar Epithelial Cells and Inflammation

AECI comprise 96% of the surface of the lung and are critical to gas exchange (Guillot et al., 2013). AECI are flat, thin cells that have substantial interaction with AMs and neighboring AECs, including those in adjacent alveoli (Guillot et al., 2013). Although AECI do not readily repair or proliferate, they can initiate the innate immune response (Yamamoto et al., 2012; Guillot et al., 2013). Pneumococcal pneumonia induces expression of CXCL5 and pattern recognition receptors TLR2 and STING (Yamamoto et al., 2012). The observed induction of innate immune responses in AECI and expression of toll-like receptors (TLR) through proinflammatory cytokines suggest a role for AECI in innate immune response initiation (Wong et al., 2012; Yamamoto et al., 2012; Guillot et al., 2013).

AECII are dynamic cells similar in number to AECI but account for far less surface area due to their cuboidal shape (Guillot et al., 2013). AECII contribute to pulmonary homeostasis by secreting surfactant and key surfactant proteins that participate in pathogen clearance (Bissonnette et al., 2020). Expression of a variety of receptors helps AECII monitor the alveolar niche and produce cytokines and other signaling molecules (Bissonnette et al., 2020). AECII are able to proliferate and act as progenitors to replace old or damaged AECI cells (Aspal and Zemans, 2020). Interaction between AM-derived immune mediators and signals from other AECs helps promote and regulate AECII proliferation and differentiation (Aspal and Zemans, 2020).

### 2.3 Macrophages of the Alveolus

Tissue-resident AMs are the predominant macrophages under physiological conditions within the lumen of the alveolus and the interstitial macrophages between alveoli (Tan and Krasnow, 2016). As the resident-dedicated phagocyte, AMs interact with both the innate and acquired immune systems, protecting the alveoli from inhaled pathogens (Trapnell and Whitsett, 2002; Evren et al., 2020). AMs interact with AECs of both types to maintain alveolar homeostasis (Bissonnette et al., 2020).

Efferocytosis is a constitutive function of macrophages required for maintaining immune homeostasis in health and during inflammatory responses (Fadok et al., 1998; McDonald et al., 1999; Ortega-Gomez et al., 2013) and is also an important antimicrobial effector mechanism to regulate intracellular pathogens (Behar et al., 2010; Martin et al., 2014). AMs are responsible for the clearance of apoptotic neutrophils through efferocytosis, the process through which apoptotic cells are removed without release of their contents (Ortega-Gomez et al., 2013; Boada-Romero et al., 2020). Defective efferocytosis results in chronic inflammation and significantly increases the likelihood of developing lung injury (Fadok et al., 1998; McDonald et al., 1999). Efferocytosis is a major contraction mechanism for the recruited macrophage pool following acute lung injury (ALI) (Janssen et al., 2011). This scenario is unique to the lungs because the alveoli and conducting airways are extraepithelial and communicate directly with the external environment. In the lung, efferocytosis can be performed by dedicated phagocytes, such as AMs and dendritic cells (DCs), and to a lesser extent by airway epithelial cells (Boada-Romero et al., 2020). In animal models, exposure to second-hand smoke impaired the ability of AMs to perform phagocytic activities, including efferocytosis. Delivery of intranasal recombinant murine GM-CSF restored AM function and improved host defense, conferring protection against pneumonia (Subramaniam et al., 2016).

Monocyte-derived AMs may be recruited and differentiated from monocytes upon lung injury or infection (Morales-Nebreda et al., 2015). Investigators have long thought that AMs originate from circulating blood monocytes, based on their observations that bone marrow-derived monocytes can replenish the AM pool after lethal irradiation or in response to injury (Morales-Nebreda et al., 2015). In response to AM injury or depletion, monocytes are recruited to the lung and differentiate into AMs (Misharin et al., 2017; Joshi et al., 2020), where they may replace the tissue-resident AM population over time (Morales-Nebreda et al., 2015). The degree to which resident AMs are replaced by persistent monocyte-derived AMs depends on several factors: the extent of AM depletion, the intensity of inflammation, and the ability of monocytes to reach the niche. During mild infections associated with a small reduction in resident macrophages, very few monocytes will engraft into the resident macrophage pool as proliferation of resident macrophages is usually sufficient (Guilliams et al., 2020).

### 2.4 Alveolar Epithelial Cell and Alveolar Macrophage Crosstalk

It is well-established that AECs and AMs communicate via autocrine and/or paracrine signaling to promote epithelial repair following injury. While particle exposure of AM-AEC co-cultures induces release of GM-CSF, TNFα, and macrophage inflammatory proteins (e.g., MIP-1β and MIP-2), AM or AEC monocultures, or fibroblast co-cultures of either AMs or AECs do not (Tao and Kobzik, 2002; Ishii et al., 2005). During pulmonary infection with *Legionella*, AECII enable communication between AMs and recruited myeloid cells (Liu et al., 2020). Infected AMs cannot produce the cytokines needed to control the infection. However, IL-1 released by infected macrophages induces AECII to produce GM-CSF, which in turn metabolically reprograms monocytes, promoting host defense (Liu et al., 2020). Epithelial cell-derived GM-CSF improves host-defense function by reprogramming monocytes to generate the cytokines needed to control infection (Liu et al., 2020); therefore, *via* AM/AECII crosstalk, GM-CSF signaling helps to orchestrate pulmonary immune response.

## 3 Pulmonary Response to Inhaled Pathogens

### 3.1 Overview of Pulmonary Acute Lung Injury and Acute Respiratory Distress Syndrome

ALI and acute respiratory distress syndrome (ARDS) have been considered events along a clinical spectrum, although the 2012 Berlin definition does not include a separate definition for the less severe ALI (Costa and Amato, 2013). Triggering events that may result in ALI and ARDS may include exposure to an inhaled chemical or physical agent, such as gases, or viral, bacterial, or fungal pathogens (Aranda-Valderrama and Kaynar, 2018). Other factors, such as genetic predispositions or comorbidities may magnify the pulmonary insult resulting from the triggering event (Aranda-Valderrama and Kaynar, 2018). ALI and ARDS are characterized by damage to the alveolar-capillary membrane resulting in excess production of inflammatory molecules, followed by noncardiogenic pulmonary edema and varying degrees of hypoxemia (Aranda-Valderrama and Kaynar, 2018; Guillamat-Prats et al., 2018). One of the major hallmarks of ALI/ARDS is AEC injury, for which the receptor for advanced glycation end products (RAGE) has been suggested as a biomarker (Uchida et al., 2006). AECII rapidly proliferate following epithelial injury, with some eventually differentiating into AECI (Fehrenbach, 2001; Herzog et al., 2008; Guillot et al., 2013). In ALI and ARDS, both AECII and AECI sustain damage, and the lack of viable AECII compromises the repair process, favoring the progression of ALI to ARDS (Pison et al., 1988; Guillamat-Prats et al., 2018). When massive inflammation cannot be controlled (as happens in ∼50% of cases), fibroblasts are activated in an attempt to repair the lung damage (Guillamat-Prats et al., 2018).

In an experimentally-induced ALI rat model, transplanted AECII reduced early inflammation, promoted recovery of lung function, and reduced mortality (Guillamat-Prats et al., 2018). Moreover, transplantation of AECII dampened inflammatory markers to control levels, demonstrating a reduction in the inflammatory response and significantly decreasing monocyte chemoattractant protein-1 concentrations in lung homogenates (Guillamat-Prats et al., 2018). The proposed mechanism by which AECII reduced injury is through release of prostaglandin E_2_ (PGE2) and surfactant protein A, thus reprogramming AMs to an anti-inflammatory phenotype (Guillamat-Prats et al., 2018). This further demonstrates the close interplay between the AECs and AMs.

### 3.2 Pulmonary Response to Infection

Upon lung infection with viruses, bacteria, or fungi, sensor cells such as AECs, AMs, and DCs initiate an immunologic response (Table 1) (Iwasaki et al., 2017). The alveolar response to challenge by pathogens is characterized by how it responds to the presence of infectious agents and how it shapes the response to the newly recruited immune cells. Given that a prolonged or exaggerated immune response can damage the respiratory tract, it is critical to closely monitor and regulate inflammation to maintain an appropriate immune response (Rubins, 2003; Newton et al., 2016).

**TABLE 1:** Immune response to common viral, bacterial, and fungal pathogens.

	Viral	Bacterial	Fungal
Common pathogen(s)	Influenza, SARS-CoV-2, RSV, human metapneumovirus (hMPV)	*Pseudomonas aeruginosa, Mycobacterium tuberculosis, Staphylococcus aureus*	*Aspergillus fumigatus, Histoplasma capsulatum*
Host response to invasion	**Influenza** • AECs are primary targets for viral replication Rosler and Herold (2016) • Pro-inflammatory mechanisms and cytopathogenic effect lead to AEC apoptosis Rosler and Herold (2016) • Viral clearance occurs through activation of immune effector cells, and epithelial repair processes including expansion of epithelial progenitor cells that reseal the epithelial layer Rosler and Herold (2016) • Supraphysiologic GM-CSF induces macrophage plasticity toward proinflammatory M1 type and is associated with reduced interferon signaling Halstead et al. (2018)	***P. aeruginosa*** • Opportunistic pathogen that tends to infect damaged epithelial cell layers, including lungs damaged by infection or mechanical ventilation, or wounds (i.e., burns); likely to cause recurrent infections (e.g., CF) Singh et al. (2015) • Innate immune response ineffective at eradicating infection, leading to host tolerance, dampened activation of host immunity, and acute pneumonia or sepsis in immunocompromised hosts Sadikot et al. (2005) ***M. tuberculosis*** • Frequently establishes stable infection upon inhalation, creating a primary intracellular niche for growth and survival Pieters (2008) • Innate resistance to *M. tuberculosis* exposure has been reported Maertzdorf et al. (2018) ***S. aureus* secondary to COVID-19 infection** • *S. aureus* is the most common cause of secondary bacterial infections in previous viral pandemics Tasher et al. (2011) • *S. aureus* bacteremia is associated with high mortality in patients with COVID-19 (54.8% 14-day mortality and 66.7% 30-day mortality post-positive blood culture) Cusumano et al. (2020) • Hospital-onset bacteremia was a significant predictor of 14-day mortality (OR 11.9; *p* = 0.01) Cusumano et al. (2020)	• AMs are one of the first lines of defense Goyal et al. (2018) • Pattern recognition receptors such as TLRs, dectin-1, dectin-2, DC-SIGN, and mannose-binding lectin identify specific fungal wall components and produce cytokines that stimulate neutrophil recruitment (the main defense mechanism) Goyal et al. (2018)
	**Primary COVID-19 infection** • AECII injury is suggested to be the main cause of COVID-19−related ARDS, while endothelial cells are less damaged Li and Ma (2020) • Th1 adaptive immune response should contribute to clearance via IFN type 1 Pelaia et al. (2020) • Some evidence of AEC activation, macrophage activation syndrome, and release of proinflammatory cytokines leading to cytokine storm syndrome and ARDS Polidoro et al. (2020) • Coinfections secondary to COVID-19 infections o COVID-19−associated pulmonary aspergillosis (CAPA) with incidence estimates of 3.8–30% Marr et al. (2021) o COVID-19−associated secondary bacterial infections associated with worse outcome severity Vaillancourt and Jorth (2020) o hMPV and RSV trigger antiviral responses that mediate clearance; Th1/Th2 skewing may dampen long term immunity and reinfection is common Gonzalez et al. (2017) o DCs become less capable of priming T cells Gonzalez et al. (2017)		**IPA** • May occur in severely immunocompromised and critically ill patients, and those with COPD Kousha et al. (2011) ***A. fumigatus*** • *A. fumigatus* causes an acute pulmonary inflammatory response that is dominated by neutrophils and to a lesser extent, macrophages Goyal et al. (2018) ***H. capsulatum*** • *H. capsulatum* is distributed worldwide and causes pulmonary and disseminated histoplasmosis, particularly in immunocompromised patients Subramanian Vignesh et al. (2013)
Role of GM-CSF	**Influenza** • AEC-derived GM-CSF is highly protective against influenza pneumonia, improving innate immune response of AMs Rosler and Herold (2016) • GM-CSF activates lung DCs Rosler and Herold, (2016) **COVID-19** • Pleiotropic; under investigation Lang et al. (2020) • Inhaled sargramostim is protective against ARDS, suggesting a potential benefit for COVID-19 Herold et al. (2014) • Inhaled sargramostim associated with boosted B-cell responses and SARS-CoV2-specific CD8^+^ T-cell responses Bosteels et al. (2021) **RSV** • RSV infection simulates expression of IFN-γ and IL-12 p40 Guerrero-Plata et al. (2005) • Overexpression of GM-CSF in the lung enhances expression of cytokines, further promoting antigen presentation and driving proliferation of antigen-presenting cells to slow viral replication Guerrero-Plata et al. (2005)	***P. aeruginosa*** • After an intratracheal inoculum with *P. aeruginosa*, GM-CSF^−/−^ mice show decreased survival compared with wild-type mice, associated with impaired AM phagocytosis, killing, and H_2_O_2_ production Ballinger et al. (2006) • GM-CSF is effective in sensitizing *P. aeruginosa* persister cells to multiple antibiotics (persister bacteria are highly tolerant to antibiotics and cause chronic infections) Choudhary et al. (2015) • GM-CSF sensitized *P. aeruginosa* biofilms to tobramycin in the presence of biofilm matrix-degrading enzymes Choudhary et al. (2015) ***M. tuberculosis*** • GM-CSF^−/−^ mice succumb to pulmonary infection by *M. tuberculosis* faster than mice with GM-CSF expression in the lungs Gonzalez-Juarrero et al. (2005) • The cell population that promotes GM-CSF−mediated innate protection against infection remains unclear Mishra et al. (2020) • GM-CSF produced by AMs may be critical for resistance against *M. tuberculosis* infection Mishra et al. (2020)	***A. fumigatus*** • Neutralizing anti-TNFα and anti-GM-CSF antibodies reduced neutrophil influx into the lung and delayed clearance of *A. fumigatus* infection in a mouse model Steinbach et al. (2003) ***H. capsulatum*** • Following *H. capsulatum* infection, GM-CSF is essential for survival in primary infection, but less critical for secondary infection Deepe et al. (1999)
	**hMPV** • Compared to RSV-infected mice, hMPV infection induced lower levels of the inflammatory cytokines IL-1, IL-6, and TNFα but was a more potent inducer of GM-CSF Guerrero-Plata et al. (2005)	***—***	***—***


AEC, alveolar epithelial cell; AECII, alveolar epithelial cell type II; AM, alveolar macrophage; ARDS, acute respiratory distress syndrome; CAPA, COVID-19−associated pulmonary aspergillosis; CF, cystic fibrosis; COPD, chronic obstructive pulmonary disease; COVID-19, coronavirus disease 2019; DC, dendritic cell; DC-SIGN, dendritic cell-specific intercellular adhesion molecule-3-grabbing non-integrin; GM-CSF, granulocyte-macrophage colony-stimulating factor; IFN-γ, interferon gamma; IPA, invasive pulmonary aspergillosis; hMPV, human metapneumovirus; OR, odds ratio; RSV, respiratory syncytial virus; SARS-CoV-2, severe acute respiratory syndrome coronavirus 2; TB, tuberculosis; TLR, toll-like receptor; TNFα, tumor necrosis factor alpha.

During the initial stages of an infection, tissue-resident innate immune cells recruit circulating neutrophils into the infected tissue, promoting recruitment of monocytes, and in turn, more neutrophils, thus potentiating a proinflammatory environment (Iwasaki et al., 2017). Neutrophil-mediated killing requires the generation and release of toxic compounds; once the infection is cleared, neutrophils undergo apoptosis within tissues and must be rapidly cleared to limit damage to healthy tissue (Ortega-Gomez et al., 2013).

AECs secrete antimicrobial peptides and proinflammatory factors in response to bacterial infection (Li et al., 2012). AMs present within the alveolar space clear airborne particles and pathogens as another roadblock to infection by secreting chemokines to attract neutrophils and monocytes. The pulmonary immune response is fine-tuned *via* communication between AMs and epithelial cells (Westphalen et al., 2014).

### 3.3 Role of Alveolar Epithelial Cells in Post-Inflammatory Pulmonary Fibrosis

Damage to the lung’s complex structure is a common characteristic of a variety of interstitial lung diseases that fall under the umbrella of pulmonary fibrosis due to an incomplete wound repair response. Chronic respiratory failure can arise due to lung function impairment from scar formation and defective gas exchange (Mulugeta et al., 2015). Infection and the corresponding inflammatory response and ARDS can result in pulmonary fibrosis (Spagnolo et al., 2020), more specifically, post-inflammatory pulmonary fibrosis which also encompasses post-inhalation, post-drowning, and ventilator-induced injury.

Recent evidence supports the “severity of epithelial injury” hypothesis originally proposed by Haschek and Witschi in 1979 in which delay of epithelial repair, not inflammation, leads to fibroblast proliferation and collagen deposition (Haschek and Witschi, 1979). Lung fibrosis is currently considered a disease of epithelial-fibroblast imbalance, suggesting a pivotal role for AEC dysfunction in the development of a fibrotic lung phenotype (Uhal and Nguyen, 2013). Substantial evidence exists that vulnerable and/or dysfunctional AECII are a pivotal player in aberrant injury/repair responses occurring in pulmonary fibrosis and other forms of fibrotic lung disease.

### 3.4. Repairing and Restoring Lung Function

The AECs are critical in normalizing lung function since they are both a major target for injury, and intrinsically involved in the repair process. Simple repair could lead to dysfunctional epithelial repopulation due to the development of scar tissue and fibrosis. Aberrant remodeling and differentiation of epithelial cells may culminate in chronic interstitial lung diseases, obstructive diseases, or the late phases of ALI/ARDS. The preferred outcome is therefore successful regeneration where fully functioning epithelial-lined airways and alveolar airspaces are created (Beers and Morrisey, 2011).

AECs respond via appropriate repair or inappropriate remodeling, which might include excessive apoptosis, epithelial-mesenchymal transition, or reprogramming to a dysfunctional state of differentiation, leading to abnormal repair and development of fibrotic scarring or abnormal pathology (Beers and Morrisey, 2011). While apoptosis is important for shaping inflammatory cell populations, inappropriate AEC apoptosis can lead to deleterious remodeling and respiratory insufficiency (Beers and Morrisey, 2011) even in the absence of inflammation (Uhal and Nguyen, 2013). AEC apoptosis is sufficient to initiate a process of fibrosis, leading to progressive scarring of the epithelium (Kim et al., 2018) mediated by activated myofibroblasts, the major contributors to fibrotic lung disease (Herzog et al., 2008). Furthermore, phagocytosis of apoptotic AECs by AMs causes a shift towards profibrotic gene expression (Kim et al., 2018).

## 4 Pulmonary Responses to Viral Infection

AMs play a critical role in controlling respiratory viral infections (RVIs) such as influenza and SARS-CoV-2 to prevent their spread. Here, we focus on the interaction between AECs and AMs in responding to these two viruses. A more comprehensive review of RVIs is denoted in Table 1.

### 4.1 Influenza Virus Infection

AECs are the primary target for human influenza infection, characterized by loss of alveolar barrier function and edema, with persistent inflammation resulting in greater capillary/alveolar leakage, culminating in severe hypoxemia and ARDS. Following viral clearance and initiation of AEC repair, local progenitor cells replicate, and the integrity of the epithelial layer is restored. Virus elimination and immune-mediated pulmonary injury are balanced by the immune response to minimize respiratory tract damage (Rosler and Herold, 2016).

### 4.2 SARS-CoV-2 Infection and COVID-19

COVID-19 is caused by SARS-CoV-2, resulting in respiratory illness including pneumonia, ARDS, respiratory failure, shock, multi-organ failure and death in severe cases (Huang et al., 2020; Prompetchara et al., 2020).

SARS-CoV-2 binds to the angiotensin-converting-enzyme 2 receptor via the viral spike protein that is cleaved by proteases to allow fusion of the viral and cellular membranes, and subsequent internalization and release of the viral RNA (Hoffmann et al., 2020). Currently, the exact mechanism by which SARS-CoV-2 injures the lung is not fully understood. The evidence points to direct viral infection of host epithelial and endothelial cells, triggering release of proinflammatory cytokines as well as dysregulation of the renin-angiotensin system (Delpino and Quarleri, 2020; Wang et al., 2020).

After the virus infects the lining of the nose, bronchi, and bronchioles, it then infects the alveoli in the distal lung, in particular AECII (Mason, 2020). AEC injury appears to be the main cause of COVID-19-related ARDS (Li and Ma, 2020). The alveoli seem to respond to SARS-CoV-2 in a biphasic manner, with initial alveolar flooding and marked hypoxia followed by an inflammatory ARDS-like response (Mason, 2020). In some patients, infection leads to the activation of AMs and the release of proinflammatory cytokines by AECs and AMs (Polidoro et al., 2020). In severe cases, tissue-resident AMs can be depleted, accompanied by an excessive influx of monocyte-derived macrophages (Liao et al., 2020).

Mounting evidence suggests that severe RVIs, especially influenza and SARS-CoV-2, can be complicated by fungal or bacterial coinfection (Table 1) (Tasher et al., 2011; Smith and McCullers, 2014; Cusumano et al., 2020; Vaillancourt and Jorth, 2020). *Aspergillus* airway overgrowth with pulmonary infection is characterized by mixed airway inflammation and bronchial invasion known as COVID-19−associated pulmonary aspergillosis (CAPA) (Marr et al., 2021). The incidence of CAPA has been reported in published studies to be 20–30% of patients with severe COVID-19 requiring mechanical ventilation. Three studies that deployed enhanced prospective screening provided incidence estimates of 14–20% (Marr et al., 2021).

Many patients with COVID-19 contract a secondary bacterial infection which frequently leads to worse outcomes (Vaillancourt and Jorth, 2020). For instance, Feng et al. reported a multicenter study of 476 patients with COVID-19 comparing outcome severity with the absence or presence of a secondary bacterial infection. Critically ill patients had the highest rate of bacterial coinfection (34.5%) compared to moderately (3.9%) and severely (8.3%) ill patients (Feng et al., 2020).

## 5 GM-CSF Regulation of Alveolar Macrophages and Pulmonary Response to Viral Infections

### 5.1 Biology of GM-CSF

GM-CSF was first characterized by Burgess et al. (1977) as a myelopoietic growth factor capable of differentiating bone marrow precursor cells into granulocytes and macrophages. It stimulates proliferation and activation of monocytes, macrophages, DCs, neutrophils, and eosinophils (Hamilton, 2015; Becher et al., 2016). GM-CSF−deficient mice primarily lack lung AMs, and mice lacking the GM-CSF receptor have markedly reduced numbers of macrophages throughout the body (Guilliams et al., 2020). GM-CSF supports the development and/or maintenance of bone marrow−derived CD103^+^ DCs (Becher et al., 2016; Lang et al., 2020) which, in mice, have been shown to be critically important for the initiation of cytotoxic CD8^+^ T-cell responses in the lung (Lang et al., 2020). GM-CSF thus serves a crucial role in normal lung health and is important for orchestrating a broad range of adaptive immune responses (Lang et al., 2020).

Following its initial discovery, GM-CSF has been identified to have a much broader role as an immune-modulating cytokine, specifically playing a critical role in AM homeostasis, lung inflammation, and immunological disease (Guillot et al., 2013; Lang et al., 2020). GM-CSF regulates the catabolism of surfactant proteins and lipids in the lung (Huffman et al., 1996). The majority of surfactant is catabolized or reutilized by AECII, and AM catabolize or phagocytose the remaining surfactant pool (Trapnell and Whitsett, 2002).

The receptor for GM-CSF is a heterodimer comprised of an α chain that is specific for GM-CSF and a signal-transducing β chain that is shared by IL-3 and IL-5 receptors (Becher et al., 2016). GM-CSF receptor activation triggers activation of JAK2 and STAT5 leading to downstream activation of PI3K signaling, as well as transcription factors, such as PU.1 and peroxisome proliferator-activated receptor-γ (PPARγ) (Figure 1B) (Bonfield et al., 2003; Becher et al., 2016; Zhan et al., 2019).

### 5.2 Sources of Pulmonary GM-CSF

GM-CSF is secreted by a variety of cells, including epithelial cells, endothelial cells, fibroblasts, and a variety of leukocytes (Shi et al., 2006; Lang et al., 2020). Production of GM-CSF in non-hematopoietic cells generally requires a stimulus (Hamilton, 2019). Certain populations of neutrophils, basophils, and eosinophils have been reported to produce GM-CSF. In addition, B cells can produce GM-CSF, particularly after activation, and GM-CSF derived from B cells in the pleural space may have an autocrine signaling role in response to lung infections and sepsis (Harris et al., 2000; Weber et al., 2014; Hamilton, 2019).

T cells, IL-1β, IL-12, and PGE2 in humans have been shown to induce GM-CSF production (Quill et al., 1989; Duhen and Campbell, 2014; Shiomi and Usui, 2015). A variety of T-cell types are important sources of GM-CSF, including Th2, CD4^+^ T cells, and Th1/17 cells (Shiomi and Usui, 2015). Some studies suggest T cells respond to CD3 blockade by secretion of GM-CSF upon stimulation with anti-CD3 (Shi et al., 2006). Th17 cells may secrete GM-CSF in response to IL-23 (Bhattacharya et al., 2015). CD4^+^ T helper cells have been identified as a cell population that primarily produce GM-CSF (Komuczki et al., 2019; Lang et al., 2020). In certain settings, transcription factors, such as nuclear factor of activated T cells (NFAT) have been shown to be required for GM-CSF production (Shang et al., 1999; Johnson et al., 2004; Shiomi and Usui, 2015). Not surprisingly, given the complexity of cytokine networks, cytokines such as IFN-γ, IL-4, IL-10, may inhibit production of GM-CSF (Shiomi and Usui, 2015). Interplay between activated T cells and macrophages results in inflammatory positive feedback loops influencing production of GM-CSF in macrophages, as well as neighboring resident tissue cells (Shi et al., 2006; Shiomi and Usui, 2015).

Alveolar epithelial cell production of GM-CSF is the major source of pulmonary GM-CSF (Gschwend et al., 2021). In lung endothelial cells and fibroblasts, TNFα and IL-1β have been shown to induce GM-CSF synthesis and production facilitating autocrine and paracrine activity in the lung (Burg et al., 2002; Fitzgerald et al., 2003; Koga et al., 2016; Hamilton, 2019). GM-CSF produced following a stimulus in endothelial cells and fibroblasts is thought to help guide tissue-invading leukocytes (Hamilton, 2019).

### 5.3 GM-CSF and Alveolar Epithelial Cells

Baseline expression of GM-CSF in AECs is low, but GM-CSF mRNA expression is constitutively present (Mir-Kasimov et al., 2012; Lang et al., 2020). However, AECII are the major non-hematopoietic, pulmonary source of GM-CSF (Gschwend et al., 2021). The inherent plasticity of AECII allows them to rapidly express GM-CSF in response to viral infection, or via induction by inflammatory cytokines, such as IL-1β, TNF-α, and in turn, expression of GM-CSF influences AECII plasticity in an autocrine fashion, inducing downstream STAT5 phosphorylation and subsequent upregulation of cyclin D1 and other cell cycle genes that regulate differentiation of AECII into AECI (Cakarova et al., 2009; Mir-Kasimov et al., 2012; Sturrock et al., 2012).

Overexpression of GM-CSF in transgenic GM-CSF knockout (GM-CSF^−/−^) mice induced AECII hyperplasia, increasing lung size and demonstrating a role for GM-CSF in the regulation of AECII proliferation and differentiation (Huffman Reed et al., 1997). Overexpression of GM-CSF also enhanced survival of mice in hyperoxia; this effect may be explained by preservation of AEC barrier function and fluid clearance, and at least in part by a reduction in hyperoxia-induced apoptosis of AECs (Paine et al., 2003).

In influenza viral infection, the proinflammatory condition prompts AECII to express GM-CSF and produces high levels of GM-CSF (Rosler and Herold, 2016). AECII also express GM-CSF receptors, facilitating both indirect and autocrine signaling that can spur AECII proliferation and decrease AEC apoptosis (Sturrock et al., 2012). GM-CSF has been shown to decrease AECII susceptibility to oxidative stress injury (Sturrock et al., 2012). GM-CSF increases Mcl-1 expression and subsequent mitochondrial cytochrome c release and induces Akt phosphorylation that was shown to protect AECs against hyperoxia (Sturrock et al., 2012).

AEC-derived GM-CSF can elicit AM activation and expansion, DC migration to lymph nodes, and T cell activation and recruitment to alveoli, resulting in viral clearance and protection from viral infection (Unkel et al., 2012; Rosler and Herold, 2016). Activated resident AMs secrete TNFα and induce AECs to express GM-CSF, initiating AEC proliferation and supporting restoration of alveolar barrier function (Cakarova et al., 2009).

### 5.4 Overview of Macrophages and GM-CSF

Macrophages are found in all mammalian tissues and develop together with the organs they populate. Through various scavenger, pattern recognition, and phagocytic receptors, macrophages sense and respond to tissue injury and invasion by infectious organisms. They play a critical role in normal tissue homeostasis, ensuring a balanced response to tissue damage. If this response is not carefully monitored and fine-tuned, inflammatory disease may result (Lavin et al., 2015). The tissue microenvironment tightly regulates the differentiation and homeostasis of macrophage cell types. Macrophage identity and function is shaped by cytokines and metabolites produced in the local environment that drive expression of specific transcription factors (Lavin et al., 2015). Examples of tissue-specific macrophages include microglial cells in the brain, splenic macrophages, Kupffer cells in the liver, and AMs in the lung (Lavin et al., 2015). Macrophages require the continuous provision of trophic factors such as IL-34, macrophage colony-stimulating factor (M-CSF) (also known as colony-stimulating factor [CSF] 1) and GM-CSF (also known as CSF2) for their development and maintenance (Lavin et al., 2015).

Surfactant accumulation impairs gas exchange, resulting in increased susceptibility to microbial infections (Rosler and Herold, 2016). Autoimmune pulmonary alveolar proteinosis (aPAP) is a rare disease characterized by the excessive accumulation of surfactant proteins within alveoli, causing progressive respiratory insufficiency. The pathogenesis associated with aPAP has been attributed to high levels of neutralizing autoantibodies against GM-CSF in the serum and bronchoalveolar lavage fluid (BALF). These autoantibodies neutralize the biologic activity of GM-CSF, impairing the maturation and phagocytosis of AMs and AM-mediated pulmonary surfactant clearance (Tazawa et al., 2010; Ohkouchi et al., 2017).

### 5.5 GM-CSF and Alveolar Macrophages

In the context of infection, GM-CSF signals growth and differentiation of myeloid cells (granulocytes and macrophages) when more of these cells are needed to fight infection, and governs emergency myelopoiesis, expanding and mobilizing progenitor myeloid cells when needed (Metcalf, 1986; Damiani et al., 2020; Lang et al., 2020). GM-CSF is essential for driving the immune functions of AMs (Figure 1B) (Rosler and Herold, 2016).

Stillborn infants lack AMs, suggesting that these cells develop postnatally, similar to observations in mice (Guilliams et al., 2013; Evren et al., 2020). This is likely because, while GM-CSF is expressed during early gestation, AM residence in the alveoli is only established at birth when the lungs are inflated, with subsequent continued maturation well into childhood (Evren et al., 2020). Transforming growth factor-beta (TGFβ) collaborates with GM-CSF to induce the transcription factor PPARγ to drive early AM differentiation and maintenance (Lambrecht, 2017; Yu et al., 2017).

GM-CSF regulates AM differentiation and innate immunity in the lung (Shibata et al., 2001). GM-CSF^−/−^ mice are prone to respiratory infections, and experimental data show that restoring GM-CSF expression reverses this susceptibility. GM-CSF^−/−^ mice have increased susceptibility to bacterial infection due to defective pulmonary clearance and abnormal cytokine production in response to infection, demonstrating the role of GM-CSF in fending off infection mediated partly by AM innate immune functions (LeVine et al., 1999). Restoring expression of GM-CSF reversed these abnormalities in AM function and reestablished expression of PU.1 (Shibata et al., 2001), a master transcription factor directing macrophage differentiation (Lloberas et al., 1999; Shibata et al., 2001).

Macrophage transcriptome and metabolomic analyses suggest that GM-CSF has pleiotropic effects on macrophage mitochondria that underlie cellular proliferation and differentiation (Wessendarp et al., 2022). Loss of GM-CSF signaling impaired amino acid biosynthesis, glycolysis, and the pentose phosphate pathway suggesting the importance for GM-CSF in facilitating mitochondrial pathways crucial to AM differentiation and proliferation (Wessendarp et al., 2022). These data provide a mitochondrial mechanism and additional understanding of how pulmonary GM-CSF regulates AM population size via STAT5 phosphorylation (Figure 1B) (Suzuki et al., 2014; Wessendarp et al., 2022).

GM-CSF helps to maintain mitochondrial structural integrity, function, and glycolytic activity (Wessendarp et al., 2022). GM-CSF receptor signaling through JAK2 and STAT5 upregulates inflammatory gene expression and results in enhanced glycolysis (Liu et al., 2020). Clearance of fatty acids and phospholipids in surfactant uses mitochondria and requires GM-CSF stimulation and regulation suggesting that GM-CSF signaling may regulate surfactant homeostasis *via* AM (Figure 1B) (Wessendarp et al., 2022).

### 5.6 GM-CSF and Alveolar Cell Crosstalk

Expression of GM-CSF by AECII occurs during late gestation after AECII fate specification and coincides with fate initiation of AM in the fetal lung (Gschwend et al., 2021). Through direct interaction with AECs, GM-CSF also improves epithelial repair processes (Cakarova et al., 2009), with preclinical data suggesting that GM-CSF may ameliorate lung injury and respiratory failure through limiting early epithelial injury and maintaining AM function (Huffman Reed et al., 1997; Paine et al., 2001; Paine et al., 2003). Recently, new data suggest that AECII-derived GM-CSF is a critical factor modulating AM development and health of mature AM (Gschwend et al., 2021).

Using single-cell RNA sequencing, 3 main macrophage populations have been identified in the interstitial space and BALF from normal lung samples: FABP4^high^, FCN1^high^, and SPP1^high^ (Morse et al., 2019). The FABP4^high^ subset represents the GM-CSF–dependent resident AMs, which are essential for the maintenance of lung homeostasis (Hajivalili et al., 2020). The FCN1^high^ subset is derived from circulating monocytes, while the origin of the SPP1^high^ subset is still unclear (Mari and Crestani, 2019). The FCN1^high^ subset displays a proinflammatory phenotype, and the SPP1^high^ subset is increased in pulmonary fibrosis and is preferentially located in fibrotic areas (Morse et al., 2019; Reyfman et al., 2019). The gene signature and localization of SPP1^high^ macrophages within fibroblast foci suggest they are the primary profibrotic macrophage in pulmonary fibrosis (Mari and Crestani, 2019; Joshi et al., 2020). Significant skewing of the pulmonary macrophage–population distribution has been demonstrated during interstitial pulmonary fibrosis and coronavirus disease 2019 (COVID-19) (Morse et al., 2019; Reyfman et al., 2019; Liao et al., 2020). As diffuse alveolar damage increases, a loss of the FABP4^high^-, GM-CSF-dependent subset occurs that may give rise to spatially restricted profibrotic niches of monocyte-derived alveolar macrophages that provide signals for fibroblast proliferation (Hajivalili et al., 2020; Joshi et al., 2020; Liao et al., 2020). Therefore, in clinical practice, there is a need to preserve as much of the GM-CSF-dependent resident population as possible to limit the degree of pulmonary fibrosis following injury to the alveolus.

**FIGURE 2:**
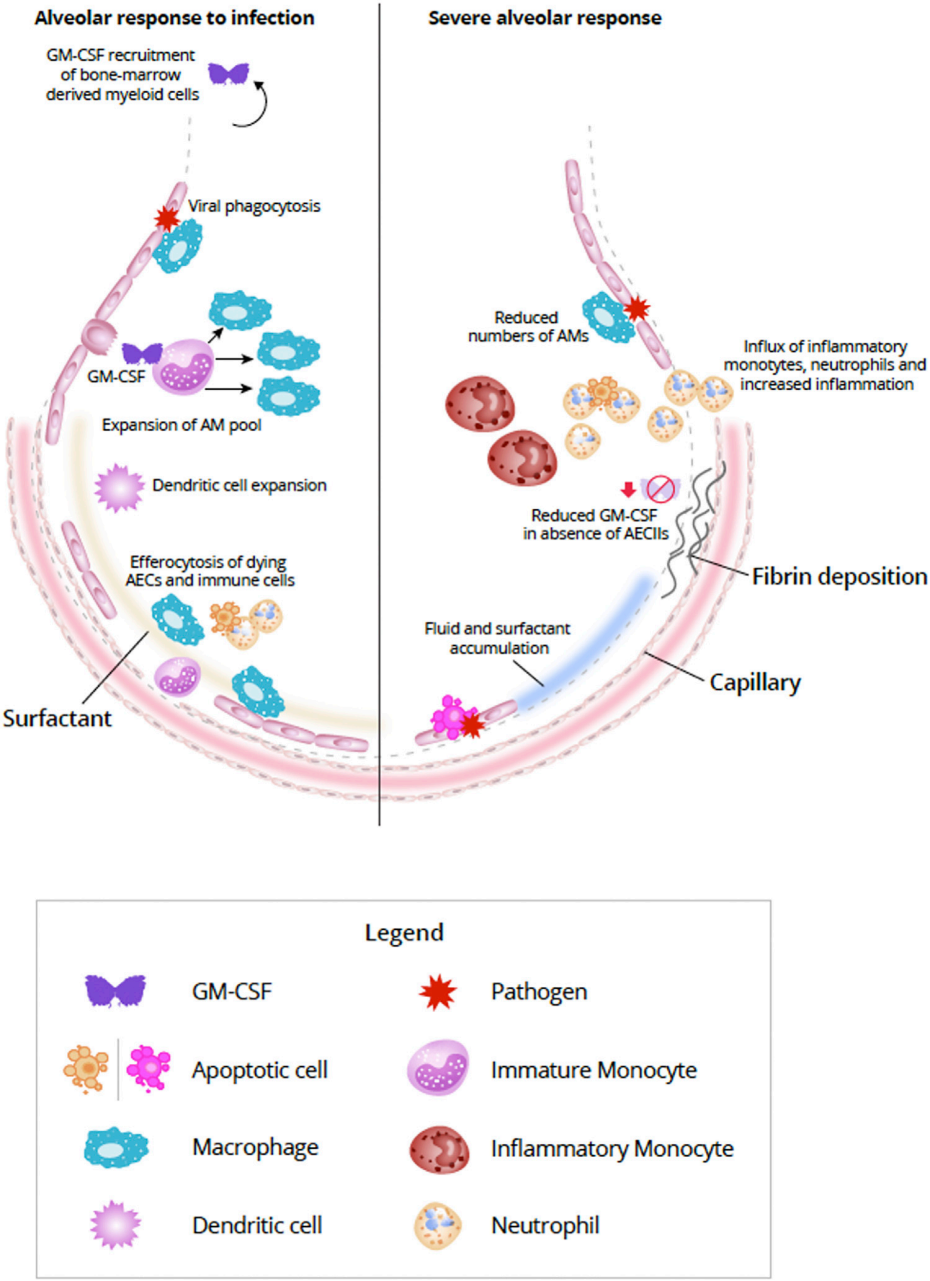
Alveolar response to viral infection (left) and severe destruction of AECs (right) (Adapted from Guillot et al., 2013). In response to lung infection, AECs and AM initiate an immunologic response (Iwasaki et al., 2017). Increased expression of GM-CSF from AECII induces differentiation of monocytes and expands the AM pool (Morales-Nebreda et al., 2015). In addition bone marrow-derived monocytes can also be summoned by alveolar-derived GM-CSF, but during mild infections proliferation of resident AM is generally sufficient (Misharin et al., 2017; Guilliams et al., 2020). In addition to viral phagocytosis, AMs also act to efferocytose apoptosing AECs and immune cells (Trapnell and Whitsett, 2002; Ortega-Gomez et al., 2013; Boada-Romero et al., 2020; Evren et al., 2020). Interaction between AECs and AMs helps to repair damage to the alveolar epithelium by expansion of AECI via AECII transdifferentiation (Aspal and Zemans, 2020). With severe destruction of AECs (right) lack of viable or sufficient AECs can compromise the repair process resulting in alveolar-capillary damage, accumulation of fluid and surfactant, increased production of inflammatory molecules, and influx of inflammatory cells, such as inflammatory monocytes and neutrophils (Pison et al., 1988; Aranda-Valderrama and Kaynar, 2018; Guillamat-Prats et al., 2018; Liao et al., 2020). Fibroblasts are activated in response to the damage and can lead to fibrotic scarring (Beers and Morrisey, 2011; Spagnolo et al., 2020). Abbreviations: AECI, alveolar epithelial cell type I; AECII, alveolar epithelial cell type II; AM, alveolar macrophage; DC, dendritic cell; GM-CSF, granulocyte-macrophage colony-stimulating factor.

GM-CSF–activated myeloid cells can secrete reactive oxygen species and express cytokines and chemokines which, in turn, attract monocytes, neutrophils, and lymphocytes (Iwasaki et al., 2017; Lang et al., 2020). GM-CSF is critically important to coordinating both innate and adaptive immune responses (Lang et al., 2020). Monocytes, granulocytes, macrophages, and dendritic cells can be modulated directly by GM-CSF via the GM-CSF receptor, thereby enabling autocrine signaling (Shi et al., 2006; Perugini et al., 2010). GM-CSF modulation of T-cell responses is thought to be indirect through the interaction of antigen-presenting cells and T cells (Shi et al., 2006). GM-CSF can enhance the ability of DCs to prime T cells during antigen-specific immune responses. A GM-CSF-producing, specific subset of CD4^+^ T helper cells has been observed which activates and recruits myeloid cells to amplify the immune response (Lang et al., 2020). Becher et al. (2016) proposed that in inflammatory conditions, lymphoid and myeloid cells communicate with each other primarily *via* GM-CSF (Figure 2).

## 6 GM-CSF and Respiratory Viral Infections

### 6.1 GM-CSF and Influenza

GM-CSF secreted by AECs can play a key role in protecting against influenza pneumonia (Rosler and Herold, 2016). The pathogen clearance of AMs in GM-CSF^−/−^ mice is impaired, reducing their resistance to influenza, while mice with elevated alveolar GM-CSF show greater AM proliferation and resistance, thus protecting against normally lethal influenza infection. Lung DCs are also critical for mediating GM-CSF−dependent protective effects. After influenza infection, pulmonary DCs activate and expand, and GM-CSF mediates their migration and antigen presentation within the draining mediastinal lymph nodes. This process is associated with improved viral clearance and antigen-specific T cell recruitment. Thus, GM-CSF is critical in mediating epithelial proliferation following lung injury, supporting repair, reestablishing barrier function, and restoring homeostasis.

Halstead et al. (2018) demonstrated GM-CSF overexpression achieved supra-physiologic levels of GM-CSF at the peak of viral replication after influenza A infection and reduced mortality and preserved lung functions in a model of GM-CSF inducible mice (WT C57BL/6 background). Transcriptome data and adoptive transfer studies suggest supraphysiologic GM-CSF in the context of active infection pushed macrophages toward the anti-inflammatory M2 type rather than the proinflammatory M1 type. Reduced interferon signaling in airways was also observed in this study, but more work is needed to elucidate the mechanisms underlying the rescue from ARDS-like illness and GM-CSF–induced plasticity in macrophage phenotype.

### 6.2 GM-CSF and Coronaviruses

Serum GM-CSF is increased in the infected tissues of patients with conditions similar to late-stage COVID-19, including ARDS (Matute-Bello et al., 1997) and cytokine release syndrome (Ahmed, 2019), as well as the SARS-CoV-2 infection itself (Hue et al., 2020). It has been suggested that SARS-CoV-1 may cause lung fibrosis through the suppression of GM-CSF (Liao et al., 2011). SARS-CoV-2 would likely impact GM-CSF expression similarly. The lung inflammation or damage biomarker Krebs von den Lungen-6 (KL-6) is principally produced by damaged or regenerating AECII (d’Alessandro et al., 2020). Recently KL-6 serum concentrations were demonstrated to be significantly higher in patients with severe COVID-19 vs. non-severe COVID-19 and healthy controls, demonstrating potential prognostic value (d’Alessandro et al., 2020).

However, lung macrophages in COVID-19 murine models are characterized by the loss of GM-CSF–mediated instruction that drives AM development, resulting in enrichment of lymphocytes, monocytes and neutrophils and depletion of macrophages in the lung (Bosteels et al., 2021). In COVID-19, instead of lung monocytes developing into AMs with sufficient GM-CSF signal, they develop into proinflammatory CD163^+^ hemophagocytic macrophages. This parallels findings from murine models demonstrating accumulation of proinflammatory monocytic or macrophagic cell populations that occur in GM-CSF–deficient conditions. Translational studies in mice suggest inhaled GM-CSF may suppress alveolar inflammation by supporting the differentiation of locally recruited monocytes to homeostatic, tissue-resident AMs.

Strategies that target the GM-CSF/GM-CSF–receptor axis have been proposed for patients with COVID-19 with and without ARDS. While GM-CSF may be beneficial for maintaining AM function during the early disease phase, neutralizing GM-CSF may reduce the primary pathology of the cytokine storm and myeloid cell-induced lung destruction in later disease stages (Lang et al., 2020). The role of inhibiting GM-CSF/GM-CSF–receptor interaction in treating patients with COVID-19 is currently under investigation in several clinical studies (ClinicalTrials.Gov, 2020a; ClinicalTrials.Gov, 2020b; ClinicalTrials.Gov, 2020e; ClinicalTrials.Gov, 2020g; ClinicalTrials.Gov, 2020h; De Luca et al., 2020; Temesgen et al., 2020). Notably, anti-GM–CSF monoclonal antibodies used to treat autoimmune disease do not appear to enter the lung, so their usefulness for treating RVI remains to be seen (Campbell et al., 2016).

### 6.3 Rationale for Sargramostim (rhu GM-CSF) for Respiratory Viral Infections

Overexpression of lung-specific GM-CSF in transgenic mice provides notable protection against various seasonal influenza strains and secondary bacterial infection through GM-CSF–dependent expansion of AMs as reviewed in Subramaniam et al. (2015). High levels of airway GM-CSF during active influenza A virus (IAV) infection confers protection from mortality from IAV and prevents the degeneration of multiple lung mechanical properties (Halstead et al., 2018). Administration of inhaled recombinant mouse GM-CSF also protected against secondary bacterial infection during influenza infection. Inhaled recombinant GM-CSF also conferred a significant survival benefit as compared to control (*p* < 0.05), whereas intraperitoneal injection did not impact survival (Huang et al., 2010; Umstead et al., 2020).

In a small clinical study, inhaled sargramostim (yeast-derived rhu GM-CSF) significantly improved oxygenation (*p* = 0.0035) without side effects in patients with severe ARDS (Herold et al., 2014), demonstrating GM-CSF is well-tolerated in the alveolar space. Given the beneficial role of inhaled recombinant GM-CSF in reducing mortality in preclinical pneumonia studies, inhaled sargramostim is under investigation to treat patients with COVID-19 (Table 2) (Huang et al., 2010; Huang et al., 2011; Bosteels et al., 2021; ClinicalTrials.Gov, 2020c; ClinicalTrials.Gov, 2020d; ClinicalTrials.Gov, 2020f; ClinicalTrials.Gov, 2021).

**TABLE 2:** Sargramostim clinical studies in COVID-19.

Name	Primary route	NCT number
Sargramostim use in COVID-19 to recover patient health (SCOPE)^a^ ClinicalTrials.Gov (2021)	Inhalation	NCT04707664
Study of sargramostim in patients with COVID-19 (iLeukPulm)^a^ ClinicalTrials.Gov (2020f)	Inhalation	NCT04411680
A phase II/III study of sargramostim in patients with Coronavirus Disease-2019 ClinicalTrials.Gov (2020c)	Inhalation	NCT04642950
Sargramostim in patients with acute hypoxic respiratory failure due to COVID-19 (SARPAC) ClinicalTrials.Gov (2020d), Bosteels et al. (2021)	Inhalation	NCT04326920
Using GM-CSF as a host directed therapeutic against COVID-19—a phase 2 investigator initiated trial ClinicalTrials.Gov (2020i)	Intravenous	NCT04400929

a Study is supported by the U.S. Department of Defense’s (DoD) Joint Program Executive Office for Chemical, Biological, Radiological and Nuclear Defense (JPEO-CBRND), as part of a contract for the advanced development and emergency use of Leukine for COVID-19 treatment (Agreement No. MCDC 2006-0120).

COVID-19, coronavirus disease 2019; GM-CSF, granulocyte-macrophage colony-stimulating factor.

### 6.4 Clinical Data for Sargramostim

A key feature of sepsis-associated immunosuppression is impaired innate and adaptive immune responses, including monocyte deactivation. A study enrolling patients with severe sepsis (*N* = 38) demonstrated that subcutaneous sargramostim restored immune function and significantly reduced mechanical ventilation time compared with placebo (148 ± 103 h vs. 207 ± 58 h; *p* = 0.0037) (Meisel et al., 2009).

Six patients with pneumonia-associated ARDS received inhaled sargramostim (Herold et al., 2014). Sargramostim significantly improved oxygenation and simplified acute physiology scores (SAPS) compared with untreated patients. This is the first evidence that sargramostim may be an effective strategy to stimulate pulmonary host defense and improve oxygenation and clinical outcomes in pneumonia-associated ARDS.

In the multi-center, open-label, randomized, controlled SARPAC trial, 81 non-ventilated hospitalized COVID-19 patients with acute hypoxemic respiratory failure (oxygen saturation below 93% on ≥ 2 L oxygen per minute or a ratio of the partial pressure of oxygen [PaO_2_] to the fraction of inspired oxygen [FiO_2_; P/F ratio] below 350 mmHg) the efficacy and safety of 5 days of inhaled sargramostim (125 μg/m^2^ twice daily) with standard of care (SOC) vs. SOC alone were assessed at day 6 (Bosteels et al., 2021). More patients in the sargramostim group experienced at least 25% improvement in oxygenation from baseline by day 6 compared with the standard of care group (62.9 vs 39.5%, *p* = 0.0459). Treatment adverse events, including signs of cytokine storm, were not different between groups. Serum concentrations of pro-inflammatory cytokines in trial patients at day 6 were not increased over baseline levels. Analysis of immune cells suggests inhaled sargramostim impacted B-cell responses and SARS-CoV2–specific CD8 T-cell responses as evidenced by significant increases in circulating switched memory B-cells and CD38^+^ HLA-DR + effector memory CD8 T-cells after 5 days of sargramostim inhalation vs SOC alone.

## 7 Discussion and Future Directions

The pleiotropic functional roles of GM-CSF in maintaining homeostasis and host defense against RVI include acting as an immunomodulator, by signaling growth and differentiation of myeloid cells when needed to fight infection, and governing myelopoiesis when macrophage niches within the lung are depleted (Damiani et al., 2020; Lang et al., 2020). GM-CSF is essential for driving the maturation and immune functions of AMs including surfactant and pathogen clearance and regulating cytokine responses to lung infection (Trapnell and Whitsett, 2002; Rosler and Herold, 2016). GM-CSF protects the host in the early phase of acute lung infection and during regeneration of the injured lung epithelium (Rosler and Herold, 2016). In the distal airways, GM-CSF promotes pathogen clearance by expanding AM or DCs, or by stimulating their host defense capacity (Rosler and Herold, 2016). GM-CSF supports the development and/or maintenance of CD103^+^ DCs, serving a crucial role in normal lung health and immune responses (Unkel et al., 2012; Rosler and Herold, 2016). Through direct interaction with AECs, GM-CSF also improves epithelial repair processes, supporting restoration of barrier function and facilitating restored homeostasis (Cakarova et al., 2009).

Given the multifaceted immunoregulatory roles of GM-CSF in lung health and host defense, careful examination and study should be taken with respect to dose, route, and timing of administration for each therapeutic approach. It is unlikely there will be a one-size-fits-all approach to therapies for patients with RVI, including COVID-19 and influenza (Hall et al., 2020; Lang et al., 2020). With the current preclinical, animal, and small clinical evidence that early administration of inhaled GM-CSF may maintain or restore surfactant levels in ARDS triggered by pulmonary infection (Matute-Bello et al., 2000; Herold et al., 2014), sargramostim may prove beneficial in monocyte-mediated trapping of the inoculum and maintaining adequate alveolar surface tension, thus preventing progression to severe forms of ARDS.

Immunomodulatory therapies that are likely to be successful in patients with COVID-19 are those tailored to the patient’s immunophenotype in real time. Clinical trials of immune modulators for the treatment of COVID-19 should include prospective immunophenotyping and/or subject stratification based on cell counts, immune function assays, cytokine levels, or other markers of inflammation (Hall et al., 2020). Clinical trials are currently underway to evaluate sargramostim in patients with COVID-19 (Table 2) (Bosteels et al., 2021; ClinicalTrials.Gov, 2020c; ClinicalTrials.Gov, 2020d; ClinicalTrials.Gov, 2020f; ClinicalTrials.Gov, 2020i; ClinicalTrials.Gov, 2021). Preclinical studies suggest that sargramostim administration may improve lung function by strengthening the alveolar wall and enhancing viral clearance. Studies of sargramostim administration in SARS-CoV-2 infection, influenza, and other RVIs, and in inhaled fungal or secondary bacterial infections provide further rationale for clinical investigation of sargramostim (Subramaniam et al., 2015).

GM-CSF may provide benefit in the earlier phases of alveolar injury/damage (e.g., influenza infection, SARS-CoV-2 infection, mechanical ventilation, and smoke inhalation) by supporting the tissue-resident AM population and maintaining the best physiologic ratio of resident AMs (Huffman Reed et al., 1997; Paine et al., 2001; Paine et al., 2003; Herold et al., 2014; Rosler and Herold, 2016; Subramaniam et al., 2016). Productive avenues for future research include the possibility of altering the ratios of FCN1^+^ and SPP1^+^/FABP4^+^ AMs to promote pathogen clearance and epithelial repair while limiting exacerbated inflammatory response and pulmonary fibrosis. The issue of appropriate timing for therapeutic modulation of viral pulmonary inflammatory responses is a matter of debate. It may be possible that altering the FCN1^+^, SPP1^+^/FABP4^+^ AM ratio in favor of maintaining a high level of FABP4^+^ cells may prevent the overwhelming proinflammatory response encountered at the later stages of COVID-19 and other RVIs (Vega et al., 2020).
